# Trajectories and Determinants of Female Sexual Function from Pregnancy to 12 Months Postpartum: Obstetric Trauma, Sleep Quality, Depressive Symptoms, and Relationship Satisfaction

**DOI:** 10.3390/jcm15062206

**Published:** 2026-03-13

**Authors:** Aris Boarta, Lavinia Stelea, Marius Lucian Craina, Bogdan Dumitriu, Ioana Denisa Socol, Madalina Ioana Sorop, Bogdan Sorop, Ileana Enatescu, Mihai Calin Bica, Adrian Gluhovschi

**Affiliations:** 1 Doctoral School, Faculty of Medicine, Victor Babes University of Medicine and Pharmacy, 300041 Timisoara, Romania; aris.boarta@umft.ro (A.B.); bogdan.dumitriu@umft.ro (B.D.); ioana.socol@umft.ro (I.D.S.); madalina.pop@umft.ro (M.I.S.); 2 Department of Obstetrics and Gynecology, Victor Babes University of Medicine and Pharmacy, 300041 Timisoara, Romania; stelea.lavinia@umft.ro (L.S.); mariuscraina@umft.ro (M.L.C.); bogdan.sorop@umft.ro (B.S.); gluhovschi.adrian@umft.ro (A.G.); 3 Discipline of Neonatology, Victor Babes University of Medicine and Pharmacy, 300041 Timisoara, Romania

**Keywords:** pregnancy, postpartum period, female sexual function, Female Sexual Function Index, sleep quality, Pittsburgh Sleep Quality Index, depressive symptoms, Patient Health Questionnaire-9, obstetric trauma, relationship satisfaction

## Abstract

**Background and objectives:** Sexual function commonly declines during late pregnancy and early postpartum, but recovery is heterogeneous and influenced by obstetric and psychosocial factors. We aimed to (i) describe longitudinal Female Sexual Function Index (FSFI) trajectories from the first trimester to 12 months postpartum and (ii) test whether sleep quality and relationship satisfaction are independently associated with sexual function at 6–12 months postpartum, beyond obstetric factors and depressive symptoms. **Methods:** In this single-center prospective cohort study, pregnant women (singleton pregnancy, ≥18 years, enrolled ≤20 gestational weeks) completed the FSFI at six timepoints: first trimester, second trimester, third trimester, 6–8 weeks postpartum, 3 months postpartum, and 6–12 months postpartum. At 6–12 months postpartum, participants also completed the Patient Health Questionnaire-9 (PHQ-9) for depressive symptoms, the Pittsburgh Sleep Quality Index (PSQI) for sleep quality, WHOQOL-BREF for quality of life, a brief body-image disturbance scale, and a 1–5 relationship satisfaction rating. Delivery was categorized as vaginal low trauma, vaginal higher trauma, or cesarean. Multivariable linear and logistic regression modeled FSFI at 6–12 months postpartum and FSFI-defined dysfunction (FSFI < 26.55). **Results:** Among 112 women, FSFI-defined dysfunction at 6–12 months postpartum affected 58.0% (65/112). Mean FSFI declined from the first trimester (26.5 ± 4.1) to 6–8 weeks postpartum (18.8 ± 4.3) and recovered by 6–12 months postpartum (25.4 ± 5.0) (time effect *p* < 0.001). Dysfunction prevalence differed by delivery group (42.2% vaginal low trauma, 63.2% cesarean, 75.9% vaginal higher trauma; *p* = 0.012). In adjusted models, worse sleep quality and higher-trauma vaginal birth increased the odds of dysfunction, whereas higher relationship satisfaction was protective. Depressive symptoms and sleep quality were independently associated with lower FSFI in linear models. **Conclusions:** Late-postpartum sexual function follows a nadir-then-recovery trajectory shaped by additive psychosocial (sleep, mood, relationship) and obstetric trauma factors, supporting multi-domain postpartum screening and targeted referral pathways.

## 1. Introduction

Female sexual function is a multidimensional component of health and quality of life, commonly conceptualized across desire, arousal, lubrication, orgasm, satisfaction, and pain. In reproductive health research, these domains are frequently quantified with the Female Sexual Function Index (FSFI), which provides both domain-level information and a total score suited for longitudinal cohort tracking [1]. Although postpartum sexual concerns are common, they remain under-discussed in routine care, and structured measurement can facilitate normalization, counseling, and timely referral [2,3].

Across pregnancy and the first postpartum year, sexual function typically shows a predictable time course: progressive decline into late pregnancy, a nadir in early postpartum, and gradual recovery thereafter [4,5]. However, recovery is heterogeneous; some women return to near pre-pregnancy levels by late postpartum, while others experience persistent difficulties in specific domains—most commonly pain, low desire, and reduced satisfaction [6,7,8,9,10]. This heterogeneity suggests that average trends may not capture clinically meaningful subgroups with distinct recovery pathways.

Obstetric factors plausibly contribute through tissue injury and pain mechanisms. Perineal trauma, episiotomy, and higher-degree lacerations have been linked to postpartum dyspareunia and delayed sexual recovery, with evidence suggesting graded relationships between trauma severity and persistence of symptoms at 6–12 months postpartum [5,8,9]. Yet obstetric exposures alone do not fully explain late-postpartum sexual outcomes, and mode of delivery has not consistently shown a protective long-term effect on global sexual function across studies [4,6].

Psychosocial context appears particularly important for distinguishing recovery from persistent dysfunction. Depressive symptoms are consistently associated with lower FSFI scores in postpartum cohorts, and relationship quality has emerged as a robust correlate of sexual satisfaction and function across the transition to parenthood [11,12,13]. In parallel, sleep disruption is near universal postpartum and may affect sexual health through fatigue, stress reactivity, mood dysregulation, dyadic functioning, and pain sensitivity [14,15]. However, sleep is often treated as a background context rather than an independent determinant in multivariable models of late-postpartum sexual function.

Accordingly, this prospective cohort study followed women from early pregnancy to 6–12 months postpartum with repeated FSFI assessment and integrated late-postpartum measurement of sleep quality and relationship satisfaction. We aimed to (i) describe FSFI trajectories across six timepoints spanning pregnancy through 12 months postpartum and (ii) evaluate whether sleep quality and relationship satisfaction are independently associated with late-postpartum sexual function beyond obstetric factors and depressive symptoms, using both continuous FSFI outcomes and clinically interpretable subgroup analyses.

## 2. Materials and Methods

### 2.1. Study Design, Setting, and Ethics

This was a single-center, prospective cohort study conducted at the Department of Obstetrics and Gynecology and affiliated outpatient services of Victor Babes University of Medicine and Pharmacy Timisoara. The protocol was designed as a pragmatic longitudinal observational study aligned with routine antenatal and postpartum follow-up schedules, enabling repeated measurement of sexual function without requiring additional clinical procedures.

Ethics approval was obtained from the institutional ethics committee, and all participants provided written informed consent. Given the intimate nature of the questionnaire content, confidentiality procedures were emphasized: surveys were completed privately without partners present, study identifiers were separated from personal identifiers, and analyses were performed on de-identified datasets.

To minimize social desirability bias, study staff were trained to provide neutral clarification of questionnaire items without prompting specific responses. Participants were explicitly informed that participation would not affect clinical care and that they could skip any question they found uncomfortable, while being encouraged to complete as much as they felt able.

Participant recruitment occurred between March 2023 and March 2024 during routine antenatal visits at the Department of Obstetrics and Gynecology and affiliated outpatient services of Victor Babeș University of Medicine and Pharmacy Timișoara (Timișoara, Romania). Postpartum follow-up and data collection continued until November 2026, with the last assessment performed at 6–12 months postpartum.

To reduce selection bias, recruitment was consecutive among eligible clinic attendees, and follow-up assessments were aligned with standard care to maximize retention. To reduce information and social desirability bias, questionnaires were self-administered privately, partners were not present, and staff provided neutral clarification without prompting responses. Exposure classification (delivery mode/trauma category) was abstracted from medical records using standardized operational definitions. To reduce confounding, multivariable models included prespecified obstetric and psychosocial covariates selected a priori based on the literature.

### 2.2. Participants, Follow-Up Schedule, and Procedures

Eligible participants were pregnant women aged ≥18 years with singleton pregnancy who enrolled at ≤20 weeks gestation during routine antenatal care. Exclusion criteria were conditions expected to strongly preclude sexual activity at baseline (medically mandated abstinence), major cognitive impairment preventing questionnaire completion, or anticipated inability to complete follow-up through 12 months postpartum.

Participants completed FSFI at six timepoints: first trimester (T1), second trimester (T2), third trimester (T3), 6–8 weeks postpartum (PP1), 3 months postpartum (PP2), and 6–12 months postpartum (PP3). This study prioritized capturing both the early postpartum nadir and later recovery period, recognizing that recovery trajectories may not be evident by the standard postpartum visit alone.

At PP3, participants completed an expanded psychosocial battery: PHQ-9 for depressive symptoms, WHOQOL-BREF domains for quality of life, a body-image disturbance scale focused on intimacy-related self-consciousness, PSQI for sleep quality, and a brief relationship satisfaction scale (1–5). Obstetric data (delivery mode and trauma category) were abstracted from the medical record using standardized definitions.

Follow-up reminders were implemented via phone or messaging to reduce attrition, and visits were aligned with routine appointments whenever possible. For PP3, in-person completion was preferred, but secure remote completion was permitted to reflect pragmatic postpartum constraints while preserving privacy.

Participants were recruited consecutively from eligible women attending antenatal care in the study setting. Study staff introduced this study in a private setting, confirmed eligibility, and obtained written informed consent. Follow-up was aligned with routine visits; participants received standardized reminders (telephone/text) before postpartum assessments. When an in-person visit was not feasible, questionnaires were completed remotely using a secure link, with instructions to complete surveys privately without partner presence.

Attrition was handled by complete-case analysis for each model; in this cohort, no enrolled participants were lost to follow-up, and all 112 completed the scheduled assessments.

The late-postpartum assessment was scheduled within a 6–12-month window to accommodate routine-care variability; when participants attended within this window, the completed assessment was treated as the PP3 timepoint.

### 2.3. Measures and Instruments

Sexual function was assessed using the Female Sexual Function Index (FSFI), a 19-item measure producing a total score from 2 to 36, where higher scores indicate better function. Although FSFI cutoffs were originally derived in non-pregnant populations, we used the widely reported threshold of FSFI < 26.55 to define “FSFI-defined sexual dysfunction” at PP3 for descriptive and comparative purposes, while analyzing FSFI primarily as a continuous outcome.

Depressive symptoms were measured with PHQ-9 (0–27), higher scores indicating greater symptom burden. Sleep quality was measured with PSQI (0–21), higher scores indicating worse sleep. Relationship satisfaction was assessed on a 1–5 scale (higher = better). Quality of life was measured using WHOQOL-BREF domain scores (0–100). Body-image disturbance was summarized on a 0–4 scale, where higher values reflect more dissatisfaction and intimacy-related self-consciousness.

Obstetric exposure was categorized into three delivery groups to balance clinical interpretability and statistical stability: (1) vaginal birth with low trauma, (2) vaginal birth with higher trauma (episiotomy and/or higher-degree lacerations as operationalized in the study dataset), and (3) cesarean delivery. Early postpartum perineal pain at PP1 was dichotomized into none/mild vs. moderate/severe. Pelvic-floor symptoms at PP3 were defined as the presence of at least one bothersome urinary or bowel symptom on a structured postpartum checklist.

An optional antenatal sexual-health counseling session (yes/no) was included as an exposure reflecting a feasible “real clinic” educational add-on rather than a formal randomized intervention. Attendance was recorded during third-trimester visits. The counseling content was standardized (normalizing common sexual changes, guidance on timing and comfort, and brief communication strategies), but uptake was voluntary.

All instruments used in this study are widely validated patient-reported measures with established feasibility and responsiveness in clinical and reproductive-health research. The FSFI has demonstrated strong psychometric performance and sensitivity to longitudinal change across reproductive life stages. Depressive symptoms (PHQ-9), sleep quality (PSQI), and quality of life (WHOQOL-BREF) were assessed using standardized instruments with established validity; Romanian-language versions have been previously used and/or validated in Romanian samples [16,17,18,19].

### 2.4. Statistical Analysis

This cohort was designed as a pragmatic longitudinal study embedded in routine care; therefore, the sample size was primarily determined by feasibility (clinic volume during the recruitment window) and the goal of achieving adequate precision for the primary descriptive endpoint (late-postpartum dysfunction prevalence) and stable estimation for prespecified multivariable models. With n = 112, the prevalence estimate for FSFI-defined dysfunction at 6–12 months postpartum has acceptable precision for cohort description, and the number of dysfunction events (n = 65) supports multivariable modeling with a limited set of a priori predictors. Missing data were minimal; participants included in the analysis completed all scheduled FSFI timepoints, and models were fit using complete-case data.

Continuous variables were summarized as mean ± standard deviation (SD) and categorical variables as counts and percentages. Normality was assessed by Shapiro–Wilk testing and distribution inspection; given the typical mild deviations and unequal variances between groups, Welch’s *t*-tests were used for most two-group comparisons of continuous outcomes, while χ^2^ tests were used for categorical comparisons (Fisher’s exact test was reserved for sparse 2 × 2 tables when applicable).

Longitudinal change in FSFI across the six timepoints was evaluated using repeated-measures ANOVA with time as the within-subject factor, reporting the overall time effect with F statistics and *p*-values. Subgroup comparisons across more than two groups used one-way ANOVA. To evaluate whether sleep quality and relationship satisfaction jointly shaped PP3 FSFI, a two-way ANOVA tested main effects and their interaction. Pearson correlations quantified associations between PP3 FSFI and psychosocial variables.

Multivariable modeling was prespecified to examine predictors beyond bivariate comparisons. Linear regression modeled PP3 FSFI as a continuous outcome, with key predictors entered simultaneously: standardized PHQ-9, PSQI, relationship satisfaction, body-image disturbance, delivery group indicators, perineal pain, pelvic-floor symptoms, counseling attendance, and age. Logistic regression modeled PP3 dysfunction (FSFI < 26.55), reporting odds ratios (ORs) with 95% confidence intervals. Two-sided *p* < 0.05 defined statistical significance.

## 3. Results

**Figure 1:**
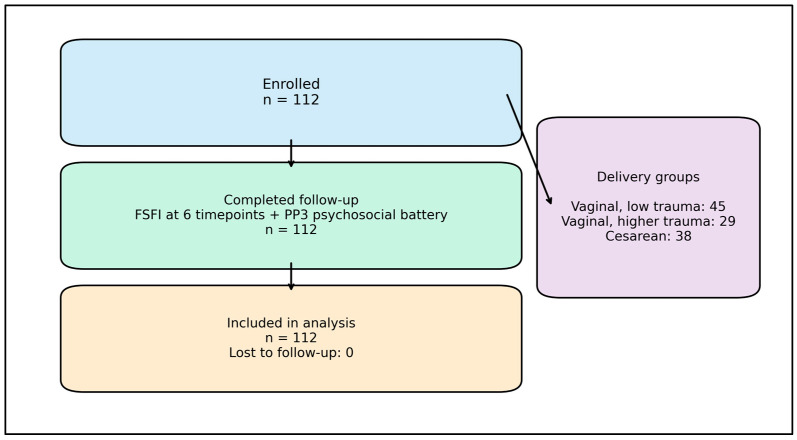
Participant flow through the cohort.

A total of 112 women were enrolled at ≤20 weeks gestation and completed the FSFI at all six scheduled timepoints (first trimester, second trimester, third trimester, 6–8 weeks postpartum, 3 months postpartum, and 6–12 months postpartum). All enrolled participants completed the late-postpartum psychosocial battery at 6–12 months postpartum, and all 112 were included in the final analysis. Delivery group distribution was vaginal low trauma (n = 45), vaginal higher trauma (n = 29), and cesarean delivery (n = 38). Participant flow from enrollment to analysis is summarized in Figure 1.

Women with PP3 dysfunction (FSFI < 26.55; n = 65) were similar to those without dysfunction (n = 47) in age (29.7 ± 4.3 vs. 28.5 ± 4.3 years; *p* = 0.172), BMI (24.3 ± 3.9 vs. 25.1 ± 3.3 kg/m^2^; *p* = 0.216), primiparity (53.8% vs. 57.4%; *p* = 0.847), perineal pain at PP1 (29.2% vs. 34.0%; *p* = 0.681), exclusive breastfeeding at PP3 (58.5% vs. 42.6%; *p* = 0.126), pelvic-floor symptoms at PP3 (33.8% vs. 31.9%; *p* = 1.000), and counseling attendance (29.2% vs. 31.9%; *p* = 0.836). However, delivery mode/trauma distribution differed significantly (*p* = 0.012), with higher-trauma vaginal birth more common among women with dysfunction (33.8% vs. 14.9%) and low-trauma vaginal birth less common (29.2% vs. 55.3%). Psychosocial and health-related measures showed clear separation: dysfunction was associated with higher depressive symptoms (PHQ-9: 8.6 ± 4.1 vs. 6.4 ± 3.3; *p* = 0.002), worse sleep (PSQI: 8.6 ± 2.8 vs. 7.0 ± 2.4; *p* = 0.002), lower relationship satisfaction (3.6 ± 0.5 vs. 3.9 ± 0.4; *p* < 0.001), and lower WHOQOL-Physical scores (66.4 ± 12.1 vs. 71.8 ± 12.0; *p* = 0.021), while WHOQOL-Psychological and body-image disturbance did not differ significantly (*p* = 0.13 and *p* = 0.485, respectively), as seen in Table 1.

FSFI total scores declined progressively across pregnancy from T1 (26.5 ± 4.1) to T2 (25.2 ± 4.2) and T3 (21.8 ± 4.5), reaching the lowest mean in early postpartum at PP1 (18.8 ± 4.3). Scores then improved at PP2 (20.6 ± 4.1) and showed substantial recovery by PP3 (25.4 ± 5.0), approaching first-trimester levels. Repeated-measures ANOVA confirmed a strong overall time effect across the six timepoints (F(5555) = 268.0, *p* < 0.001), supporting a marked late-pregnancy/early-postpartum nadir followed by gradual recovery over the first postpartum year (Table 2).

Table 3 shows a clear imbalance in late-postpartum FSFI-defined dysfunction (PP3; FSFI < 26.55) across delivery/trauma categories. Low-trauma vaginal birth had the lowest dysfunction burden (19/45, 42.2%) and the highest proportion without dysfunction (26/45, 57.8%). In contrast, higher-trauma vaginal birth clustered disproportionately in the dysfunction group (22/29, 75.9%), with only 7/29 (24.1%) classified as no dysfunction. Cesarean delivery also showed a higher dysfunction proportion than low-trauma vaginal birth (24/38, 63.2%).

**Figure 2:**
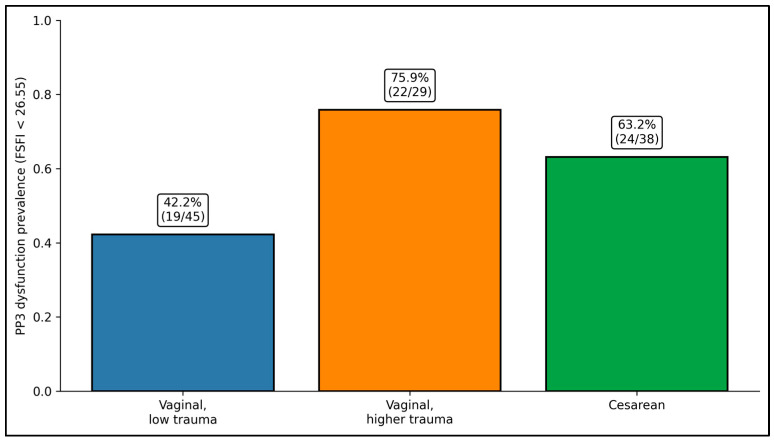
Dysfunction by delivery type.

Early postpartum sexual function (PP1) differed across delivery groups (*p* = 0.011), with the highest mean FSFI in the low-trauma vaginal group (20.3 ± 3.7; n = 45), lower scores after cesarean (18.2 ± 5.0; n = 38), and the lowest scores in the higher-trauma vaginal group (17.5 ± 3.4; n = 29). At PP3, group differences persisted (*p* = 0.027): low-trauma vaginal birth remained highest (26.7 ± 4.6), cesarean was intermediate (25.3 ± 5.6), and higher-trauma vaginal birth remained lowest (23.5 ± 4.4). Clinically, dysfunction prevalence at PP3 also varied significantly by group (*p* = 0.012), affecting 42.2% of low-trauma vaginal births, 63.2% of cesareans, and 75.9% of higher-trauma vaginal births, indicating a graded relationship between obstetric trauma category and late-postpartum sexual outcomes, as seen in Table 4 and Figure 2).

At PP3, sexual function showed strong gradients across sleep quality: mean FSFI was highest in the good-sleep tertile (27.4 ± 5.0; n = 38), slightly lower in the intermediate group (26.0 ± 5.1; n = 36), and lowest in the poor-sleep tertile (22.8 ± 3.8; n = 38), with dysfunction rates rising from 44.7% and 47.2% to 81.6%, respectively. A similar pattern was observed for relationship satisfaction: women with low relationship satisfaction had lower FSFI (23.4 ± 4.4; n = 38) and markedly higher dysfunction prevalence (84.2%) compared with moderate (25.8 ± 5.2; 45.9%) and high satisfaction tertiles (27.0 ± 4.9; 43.2%). Overall, between-tertile differences were significant for FSFI (one-way ANOVA *p* = 0.007) and for dysfunction prevalence (χ^2^ *p* < 0.001). In two-way ANOVA, both sleep (*p* < 0.001) and relationship satisfaction (*p* = 0.022) had independent main effects on PP3 FSFI, while their interaction was not significant (*p* = 0.826), suggesting additive rather than synergistic associations in this cohort (Table 5).

Correlation analyses indicated that worse depressive symptoms and poorer sleep were moderately associated with lower PP3 FSFI (PHQ-9: r = −0.4, *p* < 0.001; PSQI: r = −0.4, *p* < 0.001). Relationship satisfaction and WHOQOL-BREF physical domain showed smaller but statistically significant positive correlations with FSFI (both r = 0.2; *p* = 0.015 and *p* = 0.017, respectively), while WHOQOL-BREF psychological domain showed a similar magnitude that did not reach conventional significance (r = 0.2, *p* = 0.077). Body-image disturbance, anxiety (GAD-7), age, BMI, and breastfeeding duration showed weak and non-significant associations (|r| ≤ 0.1; *p* > 0.05 for all), suggesting that mood and sleep factors were the most prominent correlates of late-postpartum sexual function in this dataset (Table 6).

In multivariable linear regression, higher depressive symptoms and worse sleep quality were independently associated with lower PP3 FSFI (PHQ-9 per SD: β = −1.3, 95% CI −2.2 to −0.4; *p* = 0.004; PSQI per SD: β = −1.3, 95% CI −2.2 to −0.4; *p* = 0.004). Obstetric trauma also remained significant: higher-trauma vaginal delivery was associated with a 2.9-point lower FSFI compared with low-trauma vaginal delivery (β = −2.9, 95% CI −5.0 to −0.8; *p* = 0.008). Cesarean delivery showed a negative but non-significant association (β = −1.7, 95% CI −3.7 to 0.3; *p* = 0.086). Relationship satisfaction trended positively but did not reach significance (β = 0.8, 95% CI −0.1 to 1.6; *p* = 0.08), and body-image disturbance, perineal pain, pelvic-floor symptoms, counseling attendance, and age were not significant predictors (*p* ≥ 0.194). Overall model performance was moderate (R^2^ = 0.3; adjusted R^2^ = 0.2; overall *p* < 0.001), as seen in Table 7.

In logistic regression, worse sleep quality increased the odds of PP3 dysfunction (PSQI per SD: OR = 1.9, 95% CI 1.2 to 3.2; *p* = 0.009), whereas higher relationship satisfaction was protective (per SD: OR = 0.5, 95% CI 0.3 to 0.8; *p* = 0.005). Delivery group effects were substantial: higher-trauma vaginal birth was associated with markedly higher odds of dysfunction vs. low-trauma vaginal birth (OR = 5.7, 95% CI 1.7 to 19.5; *p* = 0.005), and cesarean delivery also increased odds (OR = 3.9, 95% CI 1.3 to 11.7; *p* = 0.016). Depressive symptoms showed a positive trend but did not reach statistical significance (PHQ-9 per SD: OR = 1.5, 95% CI 0.9 to 2.5; *p* = 0.097). Body-image disturbance, perineal pain, pelvic-floor symptoms, counseling attendance, and age were not significant (*p* ≥ 0.355). The overall model was significant (likelihood ratio test *p* < 0.001) with modest explanatory power (pseudo-R^2^ = 0.2), as presented in Table 8.

Table 9 quantifies the delivery-group association with PP3 dysfunction using multiple effect estimates. Compared with vaginal low-trauma birth, higher-trauma vaginal birth showed consistently elevated risk across models: unadjusted OR 4.30 (95% CI 1.56–11.87), bias-reduced OR 4.00 (1.55–10.34), unadjusted RR 1.80 (1.23–2.63), and adjusted OR 5.7 (1.7–19.5). Cesarean delivery showed a smaller and less consistent elevation: unadjusted OR 2.35 (0.96–5.77) and bias-reduced OR 2.26 (0.95–5.41) crossed/approached the null, while the adjusted OR remained significant at 3.9 (1.3–11.7).

Compared with the resilient phenotype, the pain-dominant phenotype was primarily associated with higher depressive symptoms (PHQ-9 per point: RRR = 1.1, 95% CI 1.0 to 1.2; *p* = 0.028) and higher obstetric trauma (vaginal higher trauma vs. low: RRR = 2.6, 95% CI 1.0 to 6.8; *p* = 0.047), while sleep quality and relationship satisfaction showed only borderline/non-significant associations (PSQI: *p* = 0.217; relationship satisfaction: *p* = 0.064). In contrast, the global low phenotype showed a stronger psychosocial pattern: worse sleep (PSQI per point: RRR = 1.3, 95% CI 1.1 to 1.6; *p* = 0.003) and higher depressive symptoms (PHQ-9 per point: RRR = 1.2, 95% CI 1.1 to 1.4; *p* < 0.001) increased risk, whereas greater relationship satisfaction reduced risk (RRR = 0.5, 95% CI 0.3 to 0.8; *p* = 0.006). Delivery group and pelvic-floor symptoms were not significant predictors for membership in the global-low phenotype (*p* = 0.089 to 0.65). The overall multinomial model fit was significant (likelihood ratio test *p* < 0.001) with McFadden pseudo-R^2^ = 0.2, supporting distinct predictor profiles for pain-dominant vs. global-low dysfunction phenotypes (Table 10).

**Figure 3:**
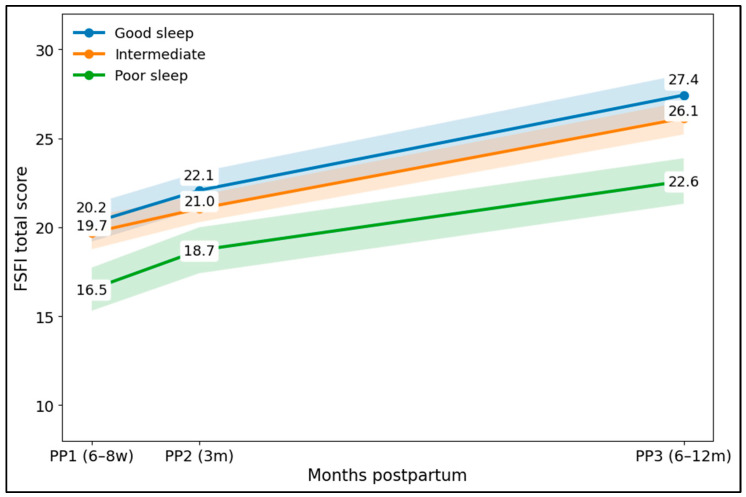
Postpartum FSFI recovery trajectories stratified by PP3 sleep tertiles (PSQI); shadows represent 95% confidence intervals.

Figure 3 shows clear separation in postpartum sexual-function recovery across sleep tertiles. Women in the good-sleep tertile had consistently higher FSFI means at each postpartum visit compared with the poor-sleep tertile, with divergence increasing over time. At PP1, mean FSFI was 20.2 (good sleep) vs. 16.5 (poor sleep), a difference of 3.7 points; at PP2, it was 22.1 vs. 18.7 (3.4 points). By PP3, mean FSFI reached 27.4 in good sleep compared with 22.6 in poor sleep, expanding the gap to 4.8 points, indicating both a lower early postpartum level and less complete recovery among those with worse sleep quality. The intermediate group tracked between these curves (PP3 26.1), supporting a graded relationship between sleep quality and FSFI recovery across the postpartum period.

**Figure 4:**
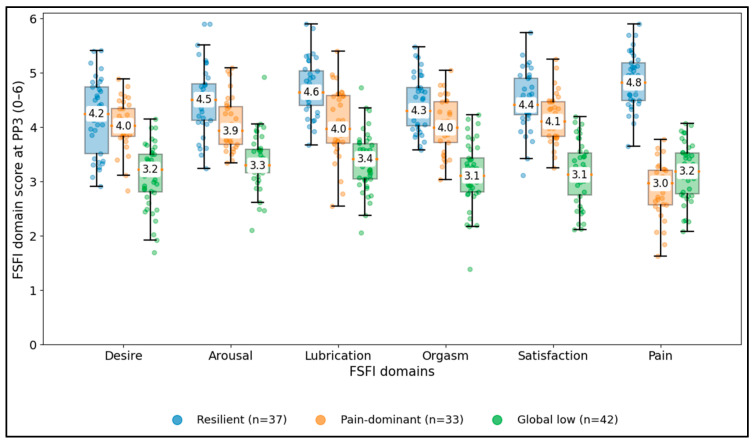
PP3 FSFI domain phenotype distributions.

Figure 4 demonstrates that postpartum sexual dysfunction can express distinct domain phenotypes rather than a uniform reduction across all FSFI components. The resilient group (n = 37) had high median domain scores across all domains, including pain (4.8) and lubrication (4.6). In contrast, the pain-dominant phenotype (n = 33) showed relatively preserved medians for desire (4.0), arousal (3.9), orgasm (4.0), and satisfaction (4.1) but a pronounced reduction in the pain domain (median 3.0), separating it from the resilient phenotype by 1.9 points in pain (4.8 vs. 3.0). The global low phenotype (n = 42) showed broadly lower medians across domains (desire 3.2, orgasm 3.1, satisfaction 3.1, pain 3.2), indicating a more generalized impairment profile. These patterns support phenotype-driven stratification (pain-focused vs. global psychosocial/biobehavioral pathways) rather than a single FSFI cutoff alone.

## 4. Discussion

### 4.1. Analysis of Findings

In this prospective cohort, FSFI scores followed a consistent nadir-then-recovery pattern: gradual decline across pregnancy, the lowest values at 6–8 weeks postpartum, and substantial improvement by 6–12 months postpartum. This trajectory aligns with prior longitudinal findings showing that sexual function is commonly most impaired in late pregnancy and early postpartum, with gradual but heterogeneous recovery over the first postpartum year. Importantly, despite recovery in mean scores by late postpartum, more than half of women met an FSFI-defined dysfunction threshold at 6–12 months, reinforcing that “average recovery” can mask a clinically meaningful subgroup with persistent impairment [20]. A distinctive contribution of this study is the integration of longitudinal FSFI trajectories with a multivariable framework that tests sleep quality as an independent determinant of late-postpartum sexual function alongside obstetric trauma, mood symptoms, and relationship satisfaction.

Our results also support the concept that postpartum sexual dysfunction is not a single uniform phenotype. The observed separation between a pain-dominant profile and a broader global-low profile is consistent with the literature emphasizing that postpartum dyspareunia and broader sexual difficulties can follow distinct pathways—one driven more by trauma-related pain and fear-avoidance and another shaped more strongly by psychosocial and biobehavioral burden (sleep disruption, mood symptoms, relational strain) [21].

Delivery mode and trauma category showed a graded association with late-postpartum outcomes: lowest dysfunction prevalence after vaginal low-trauma birth and highest after vaginal higher-trauma birth, while cesarean delivery was not protective. This pattern is consistent with studies linking perineal trauma severity and obstetric anal sphincter injury with dyspareunia and delayed sexual recovery. However, the intermediate outcomes after cesarean delivery also align with evidence that surgical recovery, postpartum fatigue, and psychosocial context can offset any reduced perineal trauma advantage, producing mixed long-term associations when global sexual function is considered. Clinically, this supports counseling that avoids framing cesarean delivery as a reliable strategy to prevent later sexual dysfunction [22].

A key contribution of this study is the demonstration that sleep quality and depressive symptoms remain independently associated with late-postpartum sexual function beyond obstetric factors. Sleep disruption may influence sexual function through multiple pathways: (i) reduced energy and increased fatigue lowering desire and arousal; (ii) increased stress reactivity and negative affect amplifying pain sensitivity and dyspareunia; (iii) hormonal and neurobiological effects of sleep loss that may influence libido and lubrication indirectly; and (iv) dyadic effects, where disrupted sleep contributes to irritability, reduced intimacy, and less effective partner communication. The additive (rather than interactive) effects of sleep quality and relationship satisfaction in our two-way model suggest that both domains contribute independently, which is clinically useful because it implies that improving either sleep or relationship context may yield benefit even if the other domain remains imperfect [23,24].

These findings support a pragmatic, measurement-based approach to postpartum sexual health. First, routine postpartum visits could incorporate brief screening of sexual function and its major correlates using feasible instruments: FSFI (or a short sexual function screener), PHQ-9, PSQI, and a brief relationship satisfaction rating. Second, stratified follow-up can be targeted to higher-risk subgroups—particularly women with higher obstetric trauma, poor sleep quality, and low relationship satisfaction—by integrating pelvic-floor assessment, dyspareunia management (lubricants, pelvic-floor physical therapy, graded exposure strategies), sleep counseling (sleep hygiene, nighttime feeding support strategies where feasible, and referral for insomnia interventions), and mental health evaluation. Third, the phenotype results suggest that domain-specific counseling may be more effective than “one-size-fits-all” reassurance: pain-dominant presentations may benefit from pelvic-floor and pain-focused care, whereas global-low profiles may require broader psychosocial intervention addressing sleep and mood with relational support [25,26].

### 4.2. Strengths and Limitations

Strengths include prospective longitudinal measurement across six timepoints and integration of validated instruments capturing sleep, mood, and quality of life alongside obstetric exposures. Limitations include the single-center design, which may limit generalizability across different sociocultural contexts and healthcare systems; the fact that several psychosocial measures were concentrated at late postpartum, which limits causal inference about time-varying mechanisms; and unmeasured confounders such as partner sexual function, contraception, prior sexual dysfunction, and breastfeeding intensity. Because sexual norms, postpartum practices, and access to pelvic-floor therapy, mental health care, and sleep support vary across settings, external validity may differ across sociocultural contexts and healthcare systems; multicenter replication would strengthen generalizability. Most psychosocial measures were assessed at late postpartum, limiting inference about temporal ordering and time-varying mediation; future studies should measure sleep, mood, and relational factors repeatedly across postpartum to better model causal pathways. Unmeasured confounding may include partner sexual function, hormonal contraception, prior sexual dysfunction, and breastfeeding intensity (beyond exclusive breastfeeding status), which should be incorporated in future prospective models. Finally, FSFI cutoffs were not developed specifically for postpartum populations and should be interpreted as a pragmatic comparative threshold rather than a diagnostic definition.

## 5. Conclusions

In this prospective cohort, sexual function declined from early pregnancy to an early-postpartum nadir and then improved substantially by 6–12 months postpartum, yet more than half of women met FSFI-defined dysfunction at late follow-up. Obstetric trauma showed a graded association with poorer outcomes, but psychosocial factors, particularly sleep quality, depressive symptoms, and relationship satisfaction, were independently linked to late-postpartum sexual function and dysfunction risk. These data support integrating multi-domain screening and targeted, modifiable interventions into routine postpartum care rather than focusing solely on delivery characteristics.

## Figures and Tables

**Table 1:** Participant characteristics by FSFI-defined sexual dysfunction at 6–12 months postpartum (PP3).

Variable	No Dysfunction (FSFI ≥ 26.55; n = 47)	Dysfunction (FSFI < 26.55; n = 65)	*p*-Value
Age (years)	28.5 ± 4.3	29.7 ± 4.3	0.172
BMI (kg/m^2^)	25.1 ± 3.3	24.3 ± 3.9	0.216
Primiparous, n (%)	27 (57.4%)	35 (53.8%)	0.847
Delivery mode group, n (%)		0.012	
Vaginal, low trauma	26 (55.3%)	19 (29.2%)	
Vaginal, higher trauma	7 (14.9%)	22 (33.8%)	
Cesarean	14 (29.8%)	24 (36.9%)	
Moderate/severe perineal pain at PP1, n (%)	16 (34.0%)	19 (29.2%)	0.681
Exclusive breastfeeding at PP3, n (%)	20 (42.6%)	38 (58.5%)	0.126
Pelvic-floor symptoms at PP3, n (%)	15 (31.9%)	22 (33.8%)	1
Antenatal sexual-health counseling attended, n (%)	15 (31.9%)	19 (29.2%)	0.836
PHQ-9 (PP3)	6.4 ± 3.3	8.6 ± 4.1	0.002
PSQI sleep score (PP3)	7.0 ± 2.4	8.6 ± 2.8	0.002
Relationship satisfaction (1–5)	3.9 ± 0.4	3.6 ± 0.5	<0.001
WHOQOL-Physical (0–100)	71.8 ± 12.0	66.4 ± 12.1	0.021
WHOQOL-Psychological (0–100)	71.1 ± 13.5	67.2 ± 13.0	0.13
Body-image disturbance (0–4)	1.6 ± 0.6	1.7 ± 0.5	0.485

BMI, body mass index; FSFI, Female Sexual Function Index; PHQ-9, Patient Health Questionnaire-9; PP1/PP3, postpartum 6–8 weeks/6–12 months; PSQI, Pittsburgh Sleep Quality Index; WHOQOL, World Health Organization Quality of Life.

**Table 2:** FSFI total scores across pregnancy and postpartum.

Timepoint	FSFI Total (Mean ± SD)
T1 (≤13 + 6 weeks)	26.5 ± 4.1
T2 (14–27 + 6 weeks)	25.2 ± 4.2
T3 (≥28 weeks)	21.8 ± 4.5
PP1 (6–8 weeks)	18.8 ± 4.3
PP2 (3 months)	20.6 ± 4.1
PP3 (6–12 months)	25.4 ± 5.0

Repeated-measures ANOVA: time effect F(5555) = 268.0, *p* < 0.001. FSFI, Female Sexual Function Index; SD, standard deviation; T1–T3, trimesters; PP1–PP3, postpartum visits.

**Table 3:** Cell counts for delivery group by late-postpartum dysfunction status (PP3; FSFI < 26.55).

Delivery Group	Dysfunction (FSFI < 26.55), n	No Dysfunction, n	Total, n
Vaginal, low trauma	19	26	45
Vaginal, higher trauma	22	7	29
Cesarean	24	14	38
Total	65	47	112

FSFI, Female Sexual Function Index; PP3, 6–12 months postpartum.

**Table 4:** Postpartum FSFI by delivery mode/trauma group (PP1 and PP3).

Delivery Group	n	FSFI PP1 (Mean ± SD)	FSFI PP3 (Mean ± SD)	PP3 Dysfunction, %
Vaginal, low trauma	45	20.3 ± 3.7	26.7 ± 4.6	42.2
Vaginal, higher trauma	29	17.5 ± 3.4	23.5 ± 4.4	75.9
Cesarean	38	18.2 ± 5.0	25.3 ± 5.6	63.2

One-way ANOVA: PP1 *p* = 0.011; PP3 *p* = 0.027.χ^2^ test (PP3 dysfunction across groups): *p* = 0.012. FSFI, Female Sexual Function Index; PP1/PP3, postpartum 6–8 weeks/6–12 months; SD, standard deviation.

**Table 5:** PP3 FSFI by sleep quality tertiles and relationship satisfaction tertiles.

Tertiles	n	FSFI PP3 (Mean ± SD)	PP3 Dysfunction, %
Sleep quality (PSQI tertiles)			
Good sleep	38	27.4 ± 5.0	44.7
Intermediate	36	26.0 ± 5.1	47.2
Poor sleep	38	22.8 ± 3.8	81.6
Relationship satisfaction tertiles			
Low relationship satisfaction	38	23.4 ± 4.4	84.2
Moderate	37	25.8 ± 5.2	45.9
High relationship satisfaction	37	27.0 ± 4.9	43.2

One-way ANOVA (FSFI): *p* = 0.007. χ^2^ (dysfunction): *p* < 0.001. Two-way ANOVA (sleep × relationship): sleep main effect *p* < 0.001; relationship main effect *p* = 0.022; interaction *p* = 0.826. FSFI, Female Sexual Function Index; PP3, 6–12 months postpartum; PSQI, Pittsburgh Sleep Quality Index; SD, standard deviation.

**Table 6:** Correlates of FSFI at 6–12 months postpartum (PP3).

Predictor	r	*p*-Value
PHQ-9 score	−0.4	<0.001
PSQI sleep score	−0.4	<0.001
Relationship satisfaction (1–5)	0.2	0.015
WHOQOL-BREF Physical	0.2	0.017
WHOQOL-BREF Psychological	0.2	0.077
Body-image disturbance (0–4)	−0.1	0.181
GAD-7 score	−0.1	0.212
Breastfeeding duration (months)	0	0.755
Age (years)	−0.1	0.131
BMI (kg/m^2^)	0.1	0.203

BMI, body mass index; FSFI, Female Sexual Function Index; GAD-7, Generalized Anxiety Disorder-7; PHQ-9, Patient Health Questionnaire-9; PP3, 6–12 months postpartum; PSQI, Pittsburgh Sleep Quality Index; WHOQOL, World Health Organization Quality of Life; BREF, brief.

**Table 7:** Multivariable predictors of PP3 FSFI.

Predictor	β	95% CI	*p*-Value
PHQ-9 (per SD)	−1.3	−2.2 to −0.4	0.004
PSQI sleep score (per SD)	−1.3	−2.2 to −0.4	0.004
Relationship satisfaction (per SD)	0.8	−0.1 to 1.6	0.08
Body-image disturbance (per SD)	−0.6	−1.4 to 0.3	0.194
Vaginal higher trauma vs. low	−2.9	−5.0 to −0.8	0.008
Cesarean vs. vaginal low trauma	−1.7	−3.7 to 0.3	0.086
Moderate/severe perineal pain (yes)	−0.2	−2.1 to 1.7	0.841
Pelvic-floor symptoms (yes)	−0.0	−1.8 to 1.8	0.994
Antenatal sexual-health counseling (yes)	0.1	−1.7 to 1.9	0.894
Age (per SD)	−0.5	−1.3 to 0.4	0.267

Model fit: R^2^ = 0.3; adjusted R^2^ = 0.2; overall model *p* < 0.001; β, unstandardized regression coefficient; CI, confidence interval; FSFI, Female Sexual Function Index; PHQ-9, Patient Health Questionnaire-9; PP3, 6–12 months postpartum; PSQI, Pittsburgh Sleep Quality Index; SD, standard deviation.

**Table 8:** Multivariable predictors of PP3 dysfunction (FSFI < 26.55).

Predictor	OR	95% CI	*p*-Value
PHQ-9 (per SD)	1.5	0.9 to 2.5	0.097
PSQI sleep score (per SD)	1.9	1.2 to 3.2	0.009
Relationship satisfaction (per SD)	0.5	0.3 to 0.8	0.005
Body-image disturbance (per SD)	1.2	0.7 to 2.0	0.503
Vaginal higher trauma vs. low	5.7	1.7 to 19.5	0.005
Cesarean vs. vaginal low trauma	3.9	1.3 to 11.7	0.016
Moderate/severe perineal pain (yes)	0.9	0.3 to 2.5	0.779
Pelvic-floor symptoms (yes)	1.5	0.5 to 4.1	0.436
Antenatal sexual-health counseling (yes)	1	0.4 to 2.8	0.99
Age (per SD)	1.2	0.8 to 2.0	0.355

Model fit: pseudo-R^2^ = 0.2; likelihood ratio test *p* < 0.001. β, unstandardized coefficient; CI, confidence interval; FSFI, Female Sexual Function Index; OR, odds ratio; PHQ-9, Patient Health Questionnaire-9; PP3, 6–12 months postpartum; PSQI, Pittsburgh Sleep Quality Index; SD, standard deviation.

**Table 9:** Delivery-group effect on PP3 dysfunction: unadjusted and sensitivity estimates (reference = vaginal low trauma).

Comparison (vs. Vaginal Low Trauma)	Unadjusted OR (95% CI)	Bias-Reduced OR (95% CI)	Unadjusted RR (95% CI)	Adjusted OR (95% CI)
Vaginal, higher trauma	4.30 (1.56–11.87)	4.00 (1.55–10.34)	1.80 (1.23–2.63)	5.7 (1.7–19.5)
Cesarean	2.35 (0.96–5.77)	2.26 (0.95–5.41)	1.50 (0.99–2.26)	3.9 (1.3–11.7)

CI, confidence interval; OR, odds ratio; PP3, 6–12 months postpartum; RR, risk ratio.

**Table 10:** Multinomial predictors of phenotype membership (RRR vs. resilient profile).

Predictor (per Unit)	Pain-Dominant RRR	95% CI	*p*-Value	Global Low RRR	95% CI	*p*-Value
PSQI (per 1 point)	1.1	0.9 to 1.3	0.217	1.3	1.1 to 1.6	0.003
PHQ-9 (per 1 point)	1.1	1.0 to 1.2	0.028	1.2	1.1 to 1.4	<0.001
Relationship satisfaction (per 1 point)	0.7	0.4 to 1.0	0.064	0.5	0.3 to 0.8	0.006
Vaginal higher trauma (vs. low)	2.6	1.0 to 6.8	0.047	2.1	0.9 to 5.1	0.089
Cesarean (vs. vaginal low trauma)	1.4	0.5 to 3.8	0.537	1.8	0.7 to 4.7	0.241
Pelvic-floor symptoms (yes)	1.8	0.7 to 4.8	0.23	1.2	0.5 to 3.1	0.65

Model fit: likelihood ratio test *p* < 0.001; McFadden pseudo-R^2^ = 0.2. CI, confidence interval; FSFI, Female Sexual Function Index; PHQ-9, Patient Health Questionnaire-9; PP3, 6–12 months postpartum; PSQI, Pittsburgh Sleep Quality Index; RRR, relative risk ratio.

## Data Availability

The data presented in this study are available on request from the corresponding author.
